# A database of freshwater macroinvertebrate occurrence records across Cuba

**DOI:** 10.1038/s41597-023-02088-0

**Published:** 2023-03-27

**Authors:** Yusdiel Torres-Cambas, Yoandri S. Megna, Juan Carlos Salazar-Salina, Yander L. Diez, Alejandro Catalá, Adrian D. Trapero-Quintana, Boris Schröder, Sami Domisch

**Affiliations:** 1 Leibniz Institute of Freshwater Ecology and Inland Fisheries, Department of Community and Ecosystem Ecology, Müggelseedamm 310, D-12489 Berlin, Germany; 2 Institute of Geoecology, Technische Universität Braunschweig, Braunschweig, D-38106 Germany; 3 Departamento de Biología y Geografía, Facultad de Ciencias Naturales y Exactas, Universidad de Oriente, Santiago de Cuba, 90500 Cuba; 4 Museum of Nature Hamburg - Zoology, Leibniz Institute for the Analysis of Biodiversity Change (LIB), Martin-Luther-King-Platz 3, D-20146 Hamburg, Germany; 5 Centre for Environmental Sciences, Research Group Zoology: Biodiversity and Toxicology, Universitaire Campus Gebouw D, B-3590 Diepenbeek, Belgium; 6 Departamento de Biología Animal y Humana, Facultad de Biología de la Universidad de la Habana, La Habana, 10400 Cuba

**Keywords:** Biodiversity, Biogeography, Freshwater ecology, Conservation biology

## Abstract

In light of the ongoing freshwater biodiversity crisis, detailed knowledge regarding the spatial distribution of freshwater species is urgently required, especially in biodiversity hotspots. Here we present a database of georeferenced occurrence records of four freshwater invertebrate taxa groups across Cuba, namely flatworms (Platyhelminthes: Tricladida), insects (Ephemeroptera, Odonata, Hemiptera, Trichoptera, Coleoptera, Diptera), crabs and shrimps (Crustacea: Decapoda), and mollusks (Mollusca). We collated the geographic occurrence information from scientific literature, unpublished field records, museum collections and online databases. The database, comprising 6292 records of 457 species at 1075 unique localities, is organized in 32 fields that contain the information about the taxonomic classification of each recorded species, the sex and life stage of collected individuals; the geographic coordinates, location, author and date of the record and a reference to the original data source. This database provides an important basis towards an improved understanding of the spatial distribution of freshwater biodiversity in Cuba.

## Background & Summary

The ongoing biodiversity crisis represents a major challenge that requires urgent countermeasures, where freely and universally accessible key scientific information is needed to address and ultimately reverse the loss of biodiversity worldwide. The spatial distribution of biodiversity can be considered such crucial information. In this regard, the lack of knowledge regarding species distributions, the so-called “Wallacean shortfall”, can be considered a bottleneck in the efforts to protect biodiversity^1^. To fill this gap, many international initiatives are promoting to digitize, geo-reference and share species distribution information that has been amassed in the past through open-access online repositories. For example, the Global Biodiversity Information Facility (GBIF), the world’s largest open-access biodiversity database, stores more than 2 billion occurrence records (August 2022, https://www.gbif.org). Despite this impressive number of records, several extremely diverse taxa groups such as freshwater invertebrates are underrepresented and there is a lack of biodiversity data from freshwater ecosystems in tropical regions^2,3^.

Freshwater macroinvertebrates consist of a heterogeneous group of organisms that includes, for example, several orders of insects, crustaceans, mollusks, annelids or flatworms. Both in terms of species richness or functional diversity and biomass, freshwater macroinvertebrates are dominant in the communities they are part of. They have in common that at least one stage of their life cycle is associated with aquatic ecosystems. This dependency on freshwater makes them particularly vulnerable to alterations in their habitats, and consequently freshwater macroinvertebrates are considered sentinels regarding the integrity of aquatic ecosystems.

**Fig. 1:**
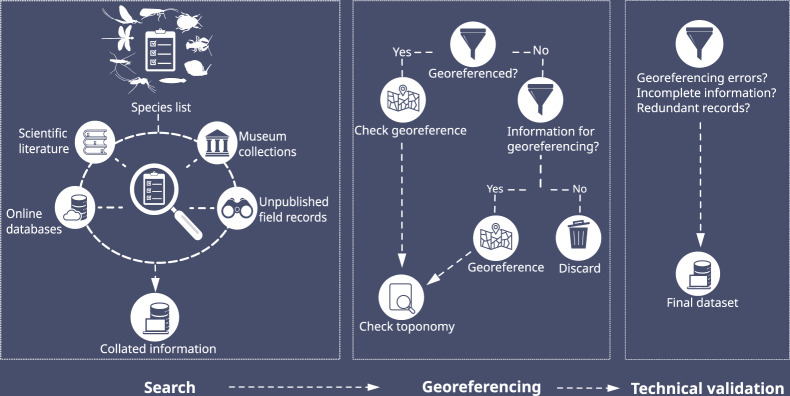
Workflow followed to compile a dataset of freshwater macroinvertebrates (i.e. Platyhelminthes, Ephemeroptera, Odonata, Hemiptera, Trichoptera, Coleoptera, Decapoda, Mollusca) from Cuba. Occurrence records were obtained from diverse sources, georeferenced and checked for possible errors and omissions.

To address the shortcoming regarding the availability of freshwater species occurrences, we present a database^4^ of freshwater macroinvertebrates for Cuba, which represents a biodiversity hotspot that has however not seen much attention to date. Our new database^4^ consists of georeferenced occurrence records of freshwater flatworms (Platyhelminthes: Tricladida), insects (Ephemeroptera, Odonata, Hemiptera, Trichoptera, Coleoptera, Diptera), crabs and shrimps (Crustacea: Decapoda), and mollusks (Mollusca) from Cuba. So far, information regarding the occurrence of freshwater species in Cuba was difficult to access by the international scientific community, since the data is scattered, or it lacks spatial information (i.e., coordinates) which is however crucial for data reusability. The Fig. 1 represents an schematic overview of the workflow followed to compile our database.

In our database, we have now included the following data sources for which the coordinates for occurrence localities were not published to date. This includes important museum collections which host specimens of Ephemeroptera^5^, Odonata^6^, Hemiptera^7^, Trichoptera^8^ and Coleoptera (Hydrophiloidea, Histeroidea)^9^, but which have not yet been digitized and georeferenced (e.g. *Museo de Historia Natural Charles Ramsden de la Torre, Universidad de Oriente; Museo de Historia Natural Felipe Poey, Universidad de La Habana*), or if digitized, the information is not hosted in online and openly accessible repositories (e.g. *Instituto de Ecología y Sistemática, La Habana*). Likewise, the database for diving beetles (Dytiscidae)^10^ with spatial explicit occurrence information, based on a revision of the literature and collections as well as field samplings conducted between 2000 and 2014, has so far not been publicly accessible. For the remaining taxa groups, maps with all known occurrence points (Ephemeroptera^5^) for each species, list of localities (Hemiptera^7^) or more general revisions of the distribution in Cuba has been published (Odonata^11,12^, Trichoptera^8^) which we collate under one comprehensive database.

The main contribution of the present database^4^ is that for the first time, occurrence records of major groups of freshwater macroinvertebrates from Cuba, with curated spatial information are compiled under the same standards (Darwin Core standards^13,14^) and made openly available according to the FAIR principles^15^. With a geographic focus on the Cuban archipelago, we expect that the database is of interest to a broader scientific community focused on the distribution and conservation of freshwater biodiversity and macroinvertebrates in particular.

## Methods

We created an updated species list^4^ (file “species_list.csv” in the database) of 590 species of tricladid flatworms (Tricladida, phylum Platyhelminthes), the insect orders Ephemeroptera (mayflies), Odonata (dragonflies and damselflies), Trichoptera (cadiflies) and freshwater Hemiptera (water bugs), Coleoptera, Decapoda (crabs and shrimps, subphylum Crustacea) and Mollusca after a revision of the literature (Table 1). We considered only binomial scientific names (genus + species), avoiding the use of subgenus and subspecies categories. This list^4^ (file “species_list.csv” in the database) was our taxonomic reference to search information from four different sources:

- Scientific literature. We conducted a search with Google Scholar using a combination of the keyword “Cuba” with “freshwater”, “Ephemeroptera”, “Odonata” or “Trichoptera” and “Cuba + freshwater” with “Platyhelminthes”, “Coleoptera”, “Heteroptera”, “Decapoda”, “Diptera”, “Mollusca”. Additionally, we consulted a collection of theses between 1980 and 2020 at Universidad de Oriente, Santiago de Cuba. We found occurrence records in 102 journal articles, 11 books and book chapters, 17 thesis dissertations and 3 scientific reports that were published between 1888 and 2022.
- Museum collections, which included the *Museo de Historia Natural Charles Ramsden de la Torre (Universidad de Oriente, Cuba)*, the *Museo de Historia Natural Felipe Poey (Universidad de La Habana, Cuba)*, and the entomological collection of the *Instituto de Ecología y Sistemática (La Habana, Cuba)*. We identified additional 33 institutions that host freshwater invertebrate specimens after the revision of the scientific literature or online databases (Table 2).

**Table 2:** List of institutions that served as a source of occurrence records of freshwater macroinvertebrates from Cuba.

Institution	Country
American Museum of Natural History, New York	USA
Colección del Departamento de Zoología, Centro Oriental de Ecosistemas y Biodiversidad, Santiago de Cuba	Cuba
Colorado Entomological Museum	USA
Departamento de Zoología, Universidad de Murcia	Spain
Florida Museum of Natural History (UF)	USA
Florida State Collection of Arthropods	USA
Forschungsinstitut und Natur-Museum Senckenberg (SMF)	Germany
Illinois Natural History Survey	USA
Instituto de Biología de la Universidad Nacional Autónoma de México	Mexico
Instituto de Ecología y Sistemática, La Habana	Cuba
Instituto Nacional de Pesquisas da Amazônia, Manaus	Brazil
Museo de Historia Natural Charles Ramsden de la Torre, Universidad de Oriente, Santiago de Cuba	Cuba
Museo de Historia Natural de Holguín	Cuba
Museo de Historia Natural Felipe Poey, Universidad de La Habana	Cuba
Museo de Zoología, Escuela de Biología, Universidad de Costa Rica, San José (UCR-MZ)	Costa Rica
Museo di Storia Naturale dell’Università di Firenze	Italy
Museum für Naturkunde, Berlin (ZMB)	Germany
Museum of Comparative Zoology, Harvard University	USA
National Museum of Natural History, Smithsonian Institution, Washington, D.C.	USA
National Museum, Prague	Czech R.
Natural History Museum of Rotterdam (NMR)	Netherlands
Naturgucker	Germany
Naturhistorisches Museum Wien	Austria
New Mexico State University (NMSU)	USA
North Carolina Museum of Natural Sciences (NCSM)	USA
Personal collection of Nico Nieser	Netherlands
Speleological Institute of Bucharest	Romania
The Field Museum of Natural History, Chicago	USA
Université Laval	Canada
University of Kansas	USA
University of Michigan Museum of Zoology (UMMZ)	USA
University of Minnesota Insect Collection (UMSP)	USA
Zoological Institute at Leningrad, Russia	Russia
Zoological Muesum La Specola, Florence	Italy
Zoological Museum of the University of Amsterdam	Netherlands
Zoologische Staatssammlung München	Germany
- Online databases. We searched in the Global Biodiversity Information Facility portal (GBIF, https://www.gbif.org, access date: 25-02-2022) and iNaturalist (https://www.inaturalist.org/, access date: 25-02-2022) using the R-package *rgbif*^16^ and *rinat*^17^ respectively. From iNaturalist, we included only “Research Grade” records in our database, after crosschecking the identification based on the photos included in this portal web. The data obtained from GBIF includes records from 26 different datasets^18^.
- Unpublished field records (n = 260), collected by the authors between 2001 and 2022.

**Table 1:** Known species richness of the taxonomic groups included in the dataset of freshwater macroinvertebrates from Cuba and literature consulted to create the species list.

Taxon	Richness	Reference
Coleoptera: Dryopidae, Dytiscidae, Elmidae, Gyrinidae, Haliplidae, Hydraenidae, Hydrochidae, Hydrophilidae, Lutrochidae, Noteridae, Scirtidae	128	^9,10,25–30,47,52–55^
Crustacea: Decapoda: Atyidae, Cambaridae, Grapsidae, Palaemonidae, Pseudothelphusidae	37	^56,57^
Diptera: Blephariceridae, Chironomidae, Culicidae, Simuliidae	95	^58,59^
Ephemeroptera	34	^5^
Hemiptera: Heteroptera: Dipsocoromorpha, Leptopodomorpha, Gerromorpha and Nepomorpha	73	^7^
Mollusca: Basommatophora, Caenogastropoda, Cycloneritomorpha, Littorinomorpha, Neritimorpha, Sorbeoconcha, Unionida, Venerida	44	^60,61^
Odonata	88	^12^
Trichoptera	89	^8^
Platyhelminthes: Tricladida	2	^62,63^

Sixteen percent (n = 1016) of records had been georeferenced by the source of information. For these we cross-checked the georeference using the standard web-client of the GEOLocate software (https://www.geo-locate.org/web/WebGeoref.aspx) and corrected if required (Table 3). We assigned coordinates to the records that lacked them only when the locality, or information such as municipality name, name of the water body, mountain range, city or town were available. In contrast, we discarded records that had only “Cuba” or a Cuban province name as spatial information. Overall, coordinate uncertainty in the database ranges from 30 m to 90892 m (median = 363 m). We added the coordinate uncertainty as an additional attribute to each record (field “coordinateUncertaintyInMeters” in the database).

**Table 3:** Occurrence records that underwent additions or updates in their coordinates or locality.

Source	Coordinates	Locality
Scientific literature	3185 (92%)	14 (0.4%)
Unpublished records	260 (100.0%)	0 (0%)
Museum collections	1545 (90%)	0 (0%)
Online databases	317 (38%)	181 (22%)
Total	5307	195

The records are organized according to their source of origin. Percent values are relative to the total records in each source class.

To find missing coordinates of the sampling sites, we conducted the following procedure. First, we searched for a record’s locality and coordinates with GEOLocate. Second, and if a locality was not found, we searched for the given locality in a Cuban 1:50 000 map (Instituto de Geodesia y Cartografía, Cuba). We assigned coordinate uncertainty in meters to all localities using GEOLocate (Table 3). We checked locality and water body names and corrected when needed, based on a gazetteer^19^ and cartographic sheets (Cuban 1:50 000 map) (Table 3). We checked the municipality and province names and updated these according to the last political-administrative organization of Cuba in 2011^20^ (http://www.onei.gob.cu/sites/default/files/dpa.pdf).

## Data Records

The database^4^ consists of 5 files: “species_list.csv” with the species list used as taxonomic reference, “occurrence_records.csv”, with occurrence records; “fields_database.docx”, that provides a complete list of the fields present in “occurrence_records.csv” and two remaining files with the scripting procedures used to query the GBIF and iNaturalist databases and for technical validation (“scr_down_o_gbif_inat.R”, “scr_tech_val.R”). The database is deposited at Figshare and can be downloaded from 10.6084/m9.figshare.21155419. In addition, the occurrence records can be queried through an interactive web application created by us with the programming language R at https://y-torres-cambas.shinyapps.io/fw_cu_dash/.

The occurrence records^4^ (“occurrence_records.csv”) are organized in 32 fields that contains information about the taxonomic classification of each recorded species, the sex and life stage of individuals, the geographic coordinates and the location (e.g. locality, water body name), the author and date of the record and the reference (i.e. URL, bibliographic reference or “newly published in this work”) to the original data source. Twenty-nine of these fields are named according to the Darwin Core standards, which is an internationally accepted vocabulary for disseminating biodiversity information^13,14^. Two of the remaining fields are based on GBIF (i.e., gbifID) and the Freshwater Core Template at the Freshwater Biodiversity Data Portal (i.e., waterBodyType, https://data.freshwaterbiodiversity.eu/). The last field consists of a sub-catchment identification number for each occurrence, extracted from the most recent high-resolution global hydrographic dataset Hydrography90m^21^. Coordinates are in decimal degrees of the World Geodetic System 1984 (WGS84).

**Fig. 2:**
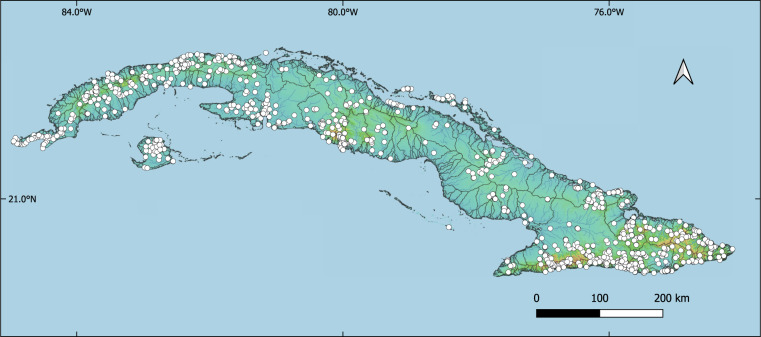
Geographic distribution of freshwater macroinvertebrates (Platyhelminthes: Tricladida, Ephemeroptera, Odonata, Hemiptera, Trichoptera, Coleoptera, Diptera, Crustacea: Decapoda, Mollusca) occurrence records across Cuba. Catchments are demarcated with black lines.

Our database^4^ (“occurrence_records.csv”) comprises a total of 6292 records of 457 species, at 1075 unique localities from 269 drainage basins of the Hydrography90m^21^ and 1080 sub-catchments (Fig. 2). Most of the records correspond to citations (i.e., a reference or citation in scholarly publications^13,14^) or preserved specimens (i.e., records from museum collections^13,14^) and, to a lesser extent, to human observations (referring to the Darwin Core standard, i.e. “an output of a human observation process” or “a record of an occurrence without physical evidence”^13,14^) (Fig. 4). The 23% (n = 1434) and 21% (n = 1324) of records have information about the sex and life stage (larvae, nymphm, exuviae, juvenile, adult, ovigerous female), respectively.

**Fig. 3:**
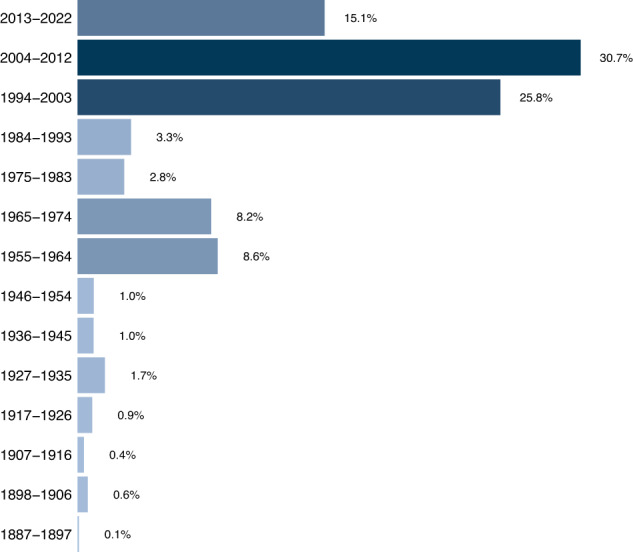
Temporal distribution of occurrence records in a dataset of freshwater macroinvertebrates from Cuba.

The records were collected between 1888 and 2022, with a highest frequency during the decades of 1960s, 1970s, 1990s, 2000s and 2010s (Fig. 3). Between 1960s–1970s, institutionalization of science in Cuba and an extensive collaboration with European scientists lead to the exploration of Cuban mountain ranges, the description of new species and data collection on species distribution, particularly in the case of freshwater invertebrates. For example, the “Cuban-Romanian Biospeleological Expeditions”, conducted during the 1960s, contributed strongly to the knowledge of Trichoptera^22^ and freshwater Decapoda^23^ and Coleoptera^24^. From 1990 to 2021 a series of studies conducted at the Department of Biology, Universidad de Oriente, Cuba or by researchers associated to this institution, on the fields of taxonomy and distribution^5,7,12,25–34^, diversity of freshwater macroinvertebrate assemblages^35–42^ and bio-monitoring^43^ in rivers of central and eastern Cuba, contributed significantly to the number of records during this period.

The 77% (n = 457) of the freshwater macroinvertebrate species listed in our species list reference^4^ (“species_list.csv”) have at least one geographic record. All taxa except Diptera have a high taxonomic representativeness, with 77% to 100% of species with at least one record (Table 4). The number of records per species ranges from 1 (for 87 species), to 189 in the Band-winged Dragonlet *Erythrodiplax umbrata* (Odonata: Libellulidae) (Fig. 5). The median number of records in each taxon is between 1 in Diptera to 23 in Odonata (Table 4). The 35% (n = 159) of the species in the file “occurrence_records.csv”^4^ have more than 10 records, however this percent value varies across taxa (Fig. 5).

**Table 4:** Descriptive statistics of the occurrence records in a dataset of freshwater macroinvertebrates from Cuba.

Taxon	N. occurrences	P. occurrences	Sp. one record	T. representativeness	Median	Med. abs. dev.
Coleoptera	1011	16%	113	88%	5	4
Decapoda	236	4%	34	92%	4	3
Diptera	90	1%	26	27%	1	0
Ephemeroptera	827	13%	29	85%	19	25
Hemiptera	465	7%	66	90%	3	3
Mollusca	481	8%	34	77%	10	11
Odonata	2675	43%	78	89%	23	20
Trichoptera	501	8%	75	84%	3	3
Tricladida	6	0.1%	2	100%	3	3

N. occurrences: number of occurrences. P. occurrences: percent of occurrences in Taxon relative to the total occurrence number in the dataset. Sp. one record: number of species with at least one occurrence record in the dataset. T. representativeness: taxonomic representativeness, percent of species with records relative to the known species richness in each taxon. Median: median occurrence records by taxon. Med. abs. dev.: median absolute deviation.

**Fig. 4:**
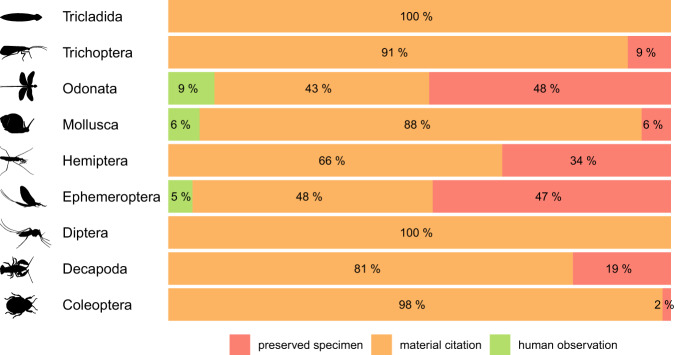
Proportion of records in different taxonomic groups according to the source of origin in a dataset of freshwater species from Cuba. The classification adopted (i.e. preserved specimen, material citation, human observation) is based on Darwin Core standards^13,14^.

**Fig. 5:**
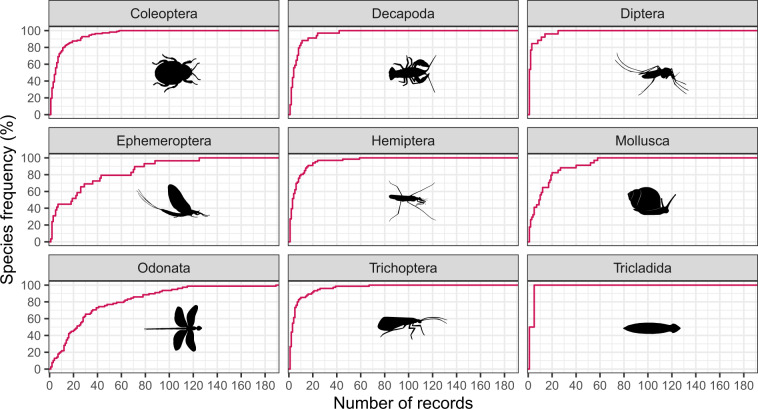
Cumulative frequency of species across the number of records per species in a dataset of freshwater macroinvertebrates from Cuba.

The order Odonata (dragonflies and damselflies) comprises most entries in the database (n = 2675, 43%, Table 4, “occurrence_records.csv”^4^). The taxonomic bias towards this order of insect can be attributed to the high detectability of Odonata in the field, and the ease to achieve a taxonomic identification of a given species. Odonata are therefore the freshwater macroinvertebrates that can be most easily detected and identified both in the laboratory and in the field in Cuba, which facilitates the recording of occurrences, including those by non-expert taxonomists and citizen scientists. For example, Odonata is the taxon with the highest proportion of records by human observation (Fig. 4), and 97% of records downloaded by us from the citizen-scientist-based online database iNaturalist correspond to Odonata (“occurrence_records.csv”^4^).

Contrary to the Odonata and the remaining taxa groups (except for Tricladida), the identification of freshwater dipterans generally requires microscope preparations and taxonomic expertise, which increases the difficulties regarding the identification to the species level. In the literature that holds information about the distribution of freshwater Diptera in Cuba, Diptera specimens, with a few exceptions^44,45^, are generally identified to genus or family^35–39^ and were therefore not included in our database^4^.

## Technical Validation

The occurrence records^4^ were screened for georeferencing errors (e.g. points in the ocean), incomplete information and redundant information with functions of the R-packages *dplyr* and *sf* (file “scr_tech_val.R”^4^). Each record must have a date, the taxonomic (species, genus, family, class, phylum) and geographic (locality, latitude, longitude, municipality and province) information and a reference to the source. A valid date must include at least the year. If two or more records of a same species had the same coordinates and date, we considered these as duplicates and only kept unique records.

## Usage Notes

The database^4^ has a high potential in spatial freshwater biodiversity analyses, biogeography and area-based conservation planning. For instance, the species geographic records can be used to model their range-wide distributions using species distribution models (SDM)^46^ with applications in the conservation of freshwater biodiversity in Cuba, for example:

- The identification of gaps in the National System of Protected Areas (e.g.^47^).
- The assessment of the conservation status of Cuban endemic species (e.g.^33^).
- The development of proactive strategies for the protection of freshwater species under future climate change (e.g.^48^).
- The improvement of the National System of Protected Areas to meet the spatial connectivity requirements of freshwater ecosystems (e.g.^49^).

We highlight that the database is welcoming future contributions. Among the taxa analyzed here, Mollusca is the taxa group for which the distribution data is not yet openly available despite that the data has been collated, digitized, georeferenced and stored systematically, such as by the Laboratory of Malacology at the Tropical Medicine Institute of Cuba who maintains a relational database with this information from 1980s to the present^50,51^.

## Data Availability

The R codes used to query the GBIF and iNaturalist databases (“scr_down_o_gbif_inat.R”) and for technical validation (“scr_tech_val.R”) are available at 10.6084/m9.figshare.21155419.
